# Drug Interactions of Metformin Involving Drug Transporter Proteins

**DOI:** 10.15171/apb.2017.062

**Published:** 2017-12-31

**Authors:** Naina Mohamed Pakkir Maideen, Abdurazak Jumale, Rajkapoor Balasubramaniam

**Affiliations:** ^1^Dubai Health Authority, Dubai, United Arab Emirates.; ^2^Department of Pharmacology, Faculty of Medicine, Sebha University, Sebha, Libya.

**Keywords:** Metformin, Drug interactions, Organic Cation Transporters, Multidrug and Toxin Extruders, Pharmacokinetic interactions

## Abstract

Metformin is a most widely used medication all around the world to treat Type 2 diabetes mellitus. It is also found to be effective against various conditions including, Prediabetes, Gestational diabetes mellitus (GDM), Polycystic Ovarian Syndrome (PCOS), Obesity, Cancer, etc. It is a cationic drug and it depends Organic Cation Transporters (OCTs) and Multidrug and Toxin Extruders (MATEs) mostly for its pharmacokinetics movement. The probability of drug interaction increases with the number of concomitant medications. This article focuses the drug interactions of metformin and most of them are linked to the inhibition of OCTs and MATEs leading to increased plasma metformin concentrations and subsequent elevation of risk of Metformin Associated Lactic Acidosis (MALA). By identifying the drugs inhibiting OCTs and MATEs, the healthcare professionals can predict the drug interactions of metformin.

## Introduction

Metformin is a popular drug and is used by millions worldwide to treat various conditions including Type 2 diabetes mellitus, Prediabetes, Gestational diabetes mellitus (GDM), Polycystic Ovarian Syndrome (PCOS), Obesity, Cancer, etc.

Metformin is primarily used as a first line drug for the treatment of type 2 diabetes mellitus in overweight patients.^1-3^ It is postulated that the antihyperglycemic action of Metformin results from decreased hepatic glucose production largely by inhibiting gluconeogenesis^4,5^ and increased glucose utilization.^6^ The activation of AMP-activated protein kinase (AMPK) by Metformin is required for the inhibition of hepatic glucose production and induction of skeletal muscle glucose uptake.^7^

## Pharmacokinetic drug interactions of Metformin

Metformin is a cation at physiological pH, as it is a strong base. Hence, the absorption, distribution and excretion of Metformin depend on the transporters such as Organic Cation Transporters (OCTs), Multidrug and Toxin Extruders (MATEs) and Plasma membrane Monoamine Transporter (PMAT).^8^ The oral absorption and hepatic uptake of Metformin are mediated possibly by Organic cation transporters (OCTs) (OCT1 and OCT3) and renal excretion of Metformin is largely mediated by Metformin transporters such as Multidrug and Toxin Extruders (MATEs) MATE1 and MATE2-k and Organic cation transporter 2 (OCT2).^9^ Metformin is not metabolized and excreted unchanged in urine^10^ and the patients with moderate and severe chronic renal impairment (CRI) should not be administered with metformin.^11^ As Metformin is not metabolized, it is not expected to be involved in many drug–drug interactions (DDIs).

Metformin use is associated to Lactic Acidosis probably due to the accumulation of lactate through the inhibition of hepatic glucose production from lactate molecules.^12^ The drugs inhibiting the Metformin transporters (MATEs and OCTs) could decrease the elimination of Metformin and increase it’s plasma concentrations leading to elevated risk of Metformin Associated Lactic Acidosis (MALA).Metformin administration should be stopped and urgent medical attention given to the patients developing first signs of MALA such as severe vomiting and diarrhea.^13^

## Interactions with Iodinated Contrast Materials (ICM)

Iodinated Contrast Materials (ICMs) used widely and successfully during many procedures including angiography, urography, etc. Administration of iodinated contrast media (CM) would result in Contrast-induced nephropathy (CIN).^14^ Hence, the risk of toxic accumulation of Metformin and subsequent Lactic Acidosis may be higher in patients taking Metformin who undergo procedures using Iodinated contrast material (ICM). The risk is further increased in patients with renal impairment and it is recommended to stop Metformin while using ICM in patients with renal impairment.^15,16^

## Interactions with acid suppressing agents

### H_2_ receptor blockers

#### Cimetidine

Cimetidine is a potent inhibitor of Multidrug and toxin extruder 1 (MATE1) of proximal tubular epithelial cells and it is a broad-spectrum inhibitor of transporters including Organic Cation Transporter 2 (OCT 2).^17,18^ Concomitant use of Metformin and Cimetidine decrease the excretion of Metformin, resulting in increased exposure of Metformin and elevated risk of Metformin Associated Lactic Acidosis (MALA).^19,20^ It is recommended to reduce the dose of Metformin when Cimetidine is co-prescribed.^21^

#### Ranitidine

Ranitidine is a potential inhibitor of Multidrug and Toxin Extruder 1 (MATE1) and hence the renal clearance of Metformin decreased.^22^

#### Famotidine

Famotidine may be suitable H2 blocker in patients taking Metformin, as it is a selective inhibitor of MATE1 and increasing the therapeutic efficacy of Metformin by significantly increasing the estimated bioavailability of Metformin. In addition, Famotidine enhances the renal clearance of Metformin compared to Cimetidine or Ranitidine which decrease it's elimination.^23^

#### Proton pump inhibitors

Proton pump inhibitors may inhibit Multidrug and toxin extruder (MATE) and OCT2 transporters and increase plasma metformin exposure.^24^ It is recommended to monitor the concomitant use of Proton pump inhibitors with Metformin.^25^

The risk of Vitamin B12 deficiency was found to be elevated by the combination of Proton pump inhibitors or H_2_ receptor blockers and Metformin. The malabsorption of vitamin B12 promoted by additive effects of Proton pump inhibitors or H_2_ receptor blockers and Metformin. Concomitant use of these drugs should be monitored for the consequences such as peripheral neuropathy and megaloblastic anemia.^26-28^ It is recommended for Vitamin B12 replacement in patients taking Metformin and PPIs/ H_2_ receptor blockers to prevent cobalamin deficiency.^29^

## Interaction with Antimicrobials

### Trimethoprim

Trimethoprim inhibits Metformin elimination moderately through the inhibition of OCTs and MATEs, but the co-administration of both the drugs should be carried out carefully in patients with renal dysfunction or patients taking higher doses of Metformin.^30^

#### Cephalexin

Cephalexin is a zwitterionic substrate of MATE1^31^ and it reduces the elimination of Metformin resulting in accumulation.^32^

#### Rifampin

Hepatic uptake of Metformin might be elevated by the administration of Rifampin due to increased expression of OCT1.^33^

#### Dolutegravir

Dolutegravir is used as the first-line antiretroviral agent in the treatment of HIV infection and it is an inhibitor of both OCT2 and MATE1 transporters within the renal tubules. Concomitant use of Dolutegravir and Metformin may result in increased adverse effects of Metformin such as hypoglycemia and GI intolerance caused by increased plasma concentrations of Metformin occurred due to the inhibition of OCT2 and MATE1 transporters. Prescribers may adjust the Metformin dose to prevent intolerable ADRs while prescribing Dolutegravir and Metformin concurrently.^34-36^

#### Pyrimethamine

Pyrimethamine is an antiparasitic drug and is used to treat toxoplasmosis and cystoisosporiasis. Pyrimethamine is an inhibitor of both OCT2 and MATE transporters.^37^ Co-administration of Pyrimethamine with Metformin results in elevated plasma concentrations due to decreased renal clearance of Metformin induced by the inhibition of OCT2 and MATE transporters by Pyrimethamine.^38^

## Interaction with Ranolazine

Ranolazine is approved to treat chronic angina. Ranolazine blocks sodium channel of pancreatic α cells and decreases electrical activity to inhibit glucagon release.^39^ The plasma concentrations of Metformin may be elevated by the co-administration of Ranolazine which may decrease the Metformin elimination through the inhibition of OCT2 transporter. This interaction is dose dependent and it is recommended that the daily dose of Metformin should not exceed 1700 mg in patients taking Ranolazine 1000 mg two times daily.^40^

## Interaction with Anticancer Drugs

### Vandetanib

Vandetanib is used in the treatment of medullary thyroid cancer. Vandetanib is a potent inhibitor of MATE1 and MATE2K transporters^41^ and its co-administration with Metformin may result in increased plasma concentrations of Metformin due to decreased elimination as it is the substrate of MATE1 and MATE2K transporters. The patients receiving the combination of Vandetanib and Metformin should be monitored carefully for Metformin toxicity.^42^

### Tyrosine kinase inhibitors

Tyrosine kinase inhibitors such as Imatinib, Nilotinib, Gefitinib, and Erlotinib may reduce the elimination of Metformin by inhibiting OCTs and MATEs transporters, at clinically relevant concentrations.^43^

## Interaction with Beta adrenergic blockers

### Atenolol

The plasma concentration of Metformin may be elevated due to reduced elimination induced by Atenolol as it reduces the renal blood flow and inhibits OCT2 competitively.^44^

### Metoprolol

The plasma concentration of Metformin can be decreased by Metoprolol by increasing the hepatic uptake of Metformin through the induction of OCT1, increasing the renal uptake of Metformin by reducing the expression of MATE1 and increasing the uptake of Metformin in thigh muscle through the induction of OCT3.^45^

## Conclusion

Most of the possible drug interactions of Metformin occur through the inhibition of OCTs and MATEs as it is not metabolized and excreted through urine as such. Iodinated Contrast Materials (ICMs) and the drugs such as Cimetidine, Ranitidine, Proton Pump Inhibitors (PPIs), Trimethoprim, Cephalexin, Dolutegravir, Pyrimethamine, Ranolazine, Vandetanib, Imatinib, Nilotinib, Gefitinib, Erlotinib and Atenolol inhibit either OCTs or MATEs or both leading to decreased elimination and increased exposure of Metformin. The risk of Metformin Associated Lactic Acidosis (MALA) enhances with the rise of plasma concentrations of Metformin. Metformin administration should be stopped and urgent medical attention given to the patients developing first signs of MALA such as severe vomiting and diarrhea. The prescribers and pharmacists should be aware of the medications inhibiting OCTs and MATEs transporters, before considering them for the patients taking Metformin.

## Ethical Issues

Not applicable.

## Conflict of Interest

Authors declare no conflict of interest in this study.
